# Transcriptional profiling reveals barcode-like toxicogenomic responses in the zebrafish embryo

**DOI:** 10.1186/gb-2007-8-10-r227

**Published:** 2007-10-25

**Authors:** Lixin Yang, Jules R Kemadjou, Christian Zinsmeister, Matthias Bauer, Jessica Legradi, Ferenc Müller, Michael Pankratz, Jens Jäkel, Uwe Strähle

**Affiliations:** 1 Institute of Toxicology and Genetics, Forschungszentrum Karlsruhe, Postfach 3640, 76021 Karlsruhe, Germany; 2 Institute for Applied Computer Science, Forschungszentrum Karlsruhe, Postfach 3640, 76021 Karlsruhe, Germany; 3 Institute for Measurement and Control Engineering, HTWK Leipzig, Postfach 30 11 66, 04251 Leipzig, Germany

## Abstract

**Background:**

Early life stages are generally most sensitive to toxic effects. Our knowledge on the action of manmade chemicals on the developing vertebrate embryo is, however, rather limited. We addressed the toxicogenomic response of the zebrafish embryo in a systematic manner by asking whether distinct chemicals would induce specific transcriptional profiles.

**Results:**

We exposed zebrafish embryos to a range of environmental toxicants and measured the changes in gene-expression profiles by hybridizing cDNA to an oligonucleotide microarray. Several hundred genes responded significantly to at least one of the 11 toxicants tested. We obtained specific expression profiles for each of the chemicals and could predict the identity of the toxicant from the expression profiles with high probability. Changes in gene expression were observed at toxicant concentrations that did not cause morphological effects. The toxicogenomic profiles were highly stage specific and we detected tissue-specific gene responses, underscoring the sensitivity of the assay system.

**Conclusion:**

Our results show that the genome of the zebrafish embryo responds to toxicant exposure in a highly sensitive and specific manner. Our work provides proof-of-principle for the use of the zebrafish embryo as a toxicogenomic model and highlights its potential for systematic, large-scale analysis of the effects of chemicals on the developing vertebrate embryo.

## Abstract 2

Microarray profiling of zebrafish embryos exposed to a range of environmental toxicants revealed distinct expression profiles for each of the toxicants tested.

## Background

Organisms are open systems that are in constant exchange with their environment. As a consequence, living systems have to adapt to environmental conditions by adjusting their physiology accordingly. Chemicals from natural sources or manmade pollution can represent rather adverse environmental conditions with a fatal outcome if the organism fails to adapt. It is a well-established fact that xenobiotics such as dioxin or cadmium can induce changes in gene expression [1-3]. The responsive genes include adaptive genes that are involved in detoxification or protection against oxidative or other cellular stresses and may also comprise genes that are directly responsible for the fatal effects of the toxicants. The early life stages of vertebrates are generally the most susceptible to adverse chemical impact [4]. Yet we do not have a detailed picture of the transcriptional response profiles of these early life stages.

There is a high demand by regulators and industry for reliable and ethically acceptable methods to evaluate the developmental toxicity of pharmaceuticals, industrial chemicals and waste products. For example, several tens of thousands of chemicals need to be assessed within the European Union REACH (Registration, Evaluation and Authorization of Chemicals) initiative for the safety testing and risk assessment of chemicals in the next years [5,6]. Cheap and reliable alternative methods are needed to cope with this enormous screening effort.

Toxicogenomics is a powerful tool for studies of toxicological mechanisms and for the detection of toxicity profiles [7] as it allows the simultaneous assessment of thousands of genes. To obtain the full potential of toxicogenomics for the evaluation of developmental toxicity, however, animal systems have to be used. The zebrafish embryo is a vertebrate system with great merits for this undertaking. The zebrafish was introduced more than two decades ago as a model to study development and neurobiology [8]. In parallel, the zebrafish embryo has evolved into a model for studies of chemical impact: it permits efficient compound screens [9] and is, for example, used in a standardized assay for sewage testing in Germany, replacing traditional toxicological tests with adult fish [10,11]. Given the experimental advantages such as small size of the embryo, cheap maintenance, availability of a genome sequence and many mutants, the zebrafish embryo is one of the most promising vertebrate systems for studies of toxicological mechanisms and toxicogenomics [12-14]. Most assays using zebrafish, however, rely on morphological endpoints, which display little discrimination between different toxicants.

Expression profiling has just recently entered zebrafish research [15-20] and only a few toxicogenomic studies exist [1,21,22]. Dioxin (TCDD) impairs fin regeneration in adult zebrafish, and expression profiling revealed TCDD-induced changes in the expression of genes involved in extracellular matrix formation [1,23]. Exposure of zebrafish to arsenic leads to changes in gene expression in adult zebrafish liver very similar to those reported for mammals, suggesting damage to protein and DNA and increased oxidative stress in the livers of arsenic-treated animals [22]. In another pilot study, zebrafish embryos were exposed to the reference compound 3,4-dichloroaniline and seven genes were significantly regulated [21].

Despite these advances, however, it is not known whether there are different responses to different toxicants and at different developmental stages. Would different toxic chemicals induce different genomic profiles, which might even be diagnostic for particular toxicants, or does the genome of the embryo respond in a general stress response. Would the sensitivity of whole-embryo exposure experiments be high enough to detect responses of genes that are restricted to small numbers of cells?

We established the toxicogenomic profiles of 11 toxicants. The gene-expression patterns induced by the 11 toxicants are related but sufficiently different to recognize toxicant-specific profiles and developmental stage-specific gene responses were also evident. Moreover, we could detect gene-expression changes at concentrations that do not have phenotypic consequences. We found synergistic effects when a mixture of compounds was applied at low doses, suggesting that the genomic response provides a more sensitive readout than morphological effects.

## Results

### Model compounds cause similar teratological and toxic effects in zebrafish embryos

We chose 11 model compounds, namely methylmercury chloride (MeHg), CdCl_2 _(Cd), PbCl2 (Pb), As_2_O_3 _(As), Aroclor 1254 (PCB), acrylamide (AA), tert-butylhydroquinone (tBHQ), 4-chloroaniline (4CA), 1,1-bis-(4-chlorophenyl)2,2,2-trichloroethane (DDT), 2,3,7,8-tetrachlorodibenzo-p-dioxin (TCDD) and valproic acid (VA). These are compounds known for their environmental toxicity [24] and VA is a teratogen and an anti-epileptic drug [25]. VA is known to inhibit histone deacetylases and Wnt signaling in mammals, thus adding an additional mode of toxic action [26].

**Figure 1:**
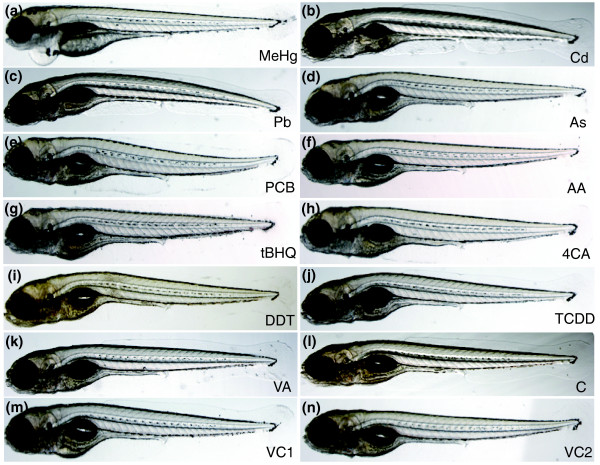
Toxicants induce similar morphological changes in 120 hpf zebrafish embryos. Embryos were treated with **(a) **methylmercury chloride (60 μg/l, MeHg); **(b) **CdCl_2 _(5 mg/l, Cd); **(c) **PbCl_2 _(2.8 mg/l, Pb); **(d) **As_2_O_3 _(79 mg/l, As); **(e) **Aroclor 1254 (33 mg/l, PCB); **(f) **acrylamide (71 mg/l, AA); **(g) **tert-butylhydroquinone (1.7 mg/l, tBHQ); **(h) **4-chloroaniline (50 mg/l, 4CA); **(i) **1,1-bis-(4-chlorophenyl)2,2,2-trichloroethane (15 mg/l, DDT); **(j) **2,3,7,8-tetrachlorodibenzo-p-dioxin (500 ng/l, TCDD); **(k) **valproic acid (50 mg/l, VA); **(l) **vehicle 1 control (VC1): embryo water alone (for Cd, MeHg, Pb, As, VA, AA treatments); **(m) **vehicle 2 control (VC2): 0.2% ethanol control (for 4CA, DDT, tBHQ, PCB); **(n) **vehicle 3 control (VC3): 0.025% DMSO, 1.4 mg/l toluene (for TCDD). Embryos showed frequently a bent body axis and developed pericardial edema upon further cultivation.

We first established exposure protocols with which one can trigger toxicogenomic alterations with high likelihood and at the same time cause only a small amount of cell death or embryo mortality. We limited the exposure time to 20-24 hours in the expectation of focusing predominantly on primary responses rather than indirect, secondary effects. Finally, we decided to carry out these assays in embryos before they begin to feed, that is, before 120 hours post-fertilization (hpf). We tested a range of toxicant concentrations to determine the one that caused a morphologically visible toxic/teratological effect in the treated embryos after exposure at 96-120 hpf (Figure 1, Table 1, Additional data file 1). We were not able to discriminate unequivocally between toxicant-specific morphological effects (see Figure 1). Frequently the tails were bent, and the animals had difficulty swimming correctly; in some instances they developed pericardial edema (see Figure 1a). Vehicle-treated embryos did not show alterations (see Figure 1l-n) or did so only at very low frequency. Cell death as monitored by acridine orange staining was not, or only rarely, obvious immediately after treatment when animals were sacrificed for microarray analysis.

**Table 1:** Summary of microarray experiments

Toxicants	Stage	Concentration	Arrays
4CA	24 hpf	15 ppm 15 mg/l 118 μM	8 (3)
	48 hpf	50 ppm 50 mg/l 390 μM	6 (3)
	120 hpf	50 ppm 50 mg/l 390 μM	8 (3)
	120 hpf	25 ppm 25 mg/l 195 μM	4 (1)
	120 hpf	5 ppm 5 mg/l 39 μM	4 (1)
	120 hpf	0.5 ppm 0.5 mg/l 3.9 μM	4 (1)
DDT	24 hpf	5 ppm 5 mg/l 14 μM	6 (3)
	48 hpf	15 ppm 15 mg/l 42 μM	6 (2)
	120 hpf	15 ppm 15 mg/l 42 μM	8 (3)
	120 hpf	1.5 ppm 1.5 mg/l 4.2 μM	4 (1)
	120 hpf	0.15 ppm 0.15 mg/l 0.42 μM	4 (1)
Cd	24 hpf	0.5 ppm 0.5 mg/l 2.7 μM	8 (4)
	48 hpf	5 ppm 5 mg/l 27 μM	8 (3)
	120 hpf	5 ppm 5 mg/l 27 μM	8 (3)
	120 hpf	2.5 ppm 2.5 mg/l 13.5 μM	4 (2)
	120 hpf	0.5 ppm 0.5 mg/l 2.7 μM	4 (2)
	120 hpf	50 ppb 50 μg/l 0.27 μM	4 (2)
TCDD	24 hpf	150 ppt 150 ng/l 0.47 nM	8 (3)
	48 hpf	500 ppt 500 ng/l 1.6 nM	4 (2)
	120 hpf	500 ppt 500 ng/l 1.6 nM	8 (3)
	120 hpf	250 ppt 250 ng/l 0.8 nM	4 (1)
	120 hpf	50 ppt 50 ng/l 0.16 nM	4 (2)
VA	24 hpf	15 ppm 15 mg/l 12.9 μM	8 (3)
	48 hpf	50 ppm 50 mg/l 43 μM	8 (3)
	120 hpf	50 ppm 50 mg/l 43 μM	8 (3)
	120 hpf	25 ppm 25 mg/l 21.5 μM	4 (1)
	120 hpf	5 ppm 5 mg/l 4.3 μM	4 (1)
	120 hpf	0.5 ppm 0.5 mg/l 0.43 μM	4 (1)
MeHg	24 hpf	50 ppb 50 μg/l 0.20 μM	8 (3)
	48 hpf	60 ppb 60 μg/l 0.24 μM	6 (2)
	120 hpf	60 ppb 60 μg/l 0.24 μM	10 (3)
	120 hpf	30 ppb 30 μg/l 0.12 μM	4 (2)
	120 hpf	6 ppb 6 μg/l 0.024 μM	4 (2)
As	120 hpf	79 ppm 79 mg/l 400 μM	8 (3)
	120 hpf	7.9 ppm 7.9 mg/l 40 μM	4 (1)
Pb	120 hpf	2.8 ppm 2.8 mg/l 10 μM	8 (3)
	120 hpf	0.28 ppm 0.28 mg/l 1 μM	4 (1)
PCB	120 hpf	33 ppm 33 mg/l 100 μM	8 (3)
AA	120 hpf	71 ppm 71 mg/l 1 mM	8 (3)
tBHQ	120 hpf	1.7 ppm 1.7 mg/l 10 μM	8 (3)
Mixture	120 hpf	Pb 1 μM, Cd 0.27 μM, As 40 μM, Hg 0.024 μM	6 (3)

Embryos were either treated from 4 to 24 (24 hpf) or from 24 to 48 hpf (48 hpf) or from 96 to 120 hpf (5 days). Arrays, total number of microarray hybridizations. Numbers in brackets indicate the number of independent biological repeats. 4CA, 4-chloroaniline; DDT, 1,1-bis-(4-chlorphenyl)-2,2,2-trichlorethane; Cd, cadmium chloride; TCDD, 2,3,7,8-tetrachlorodibenzo-p-dioxin; VA, valproic acid; MeHg, methylmercury chloride; As, arsenic (III) oxide; Pb, lead (II) chloride; AA, acrylamide, PCB, Aroclor 1254; tBHQ, tert-butylhydroquinone.

Between 96 and 120 hpf organogenesis has proceeded so far that the animals feed for the first time [8], marking the end of the embryonic stage. At this stage, gut, liver, pancreas, nervous system, musculature and the cardiovascular system are assumed to reflect adult physiology in many respects, including the response to toxicants. Younger embryonic stages are likely to have different responses to the toxicants. We therefore included two more stages in our initial experiments. The 4-24 hpf treatment covers late blastula, gastrula and segmentation stages, during which the overall body plan is laid down [8]. The treatment phase between 24 and 48 hpf coincides with the onset of organogenesis [8]. Early embryonic stages appear more sensitive to toxicant exposure than the older embryos (compare the 24-48 hpf and 96-120 hpf treatment groups in Table 1). The concentrations of the toxicants were adjusted accordingly (see Table 1).

### Stage-specific toxicogenomic responses

**Figure 2:**
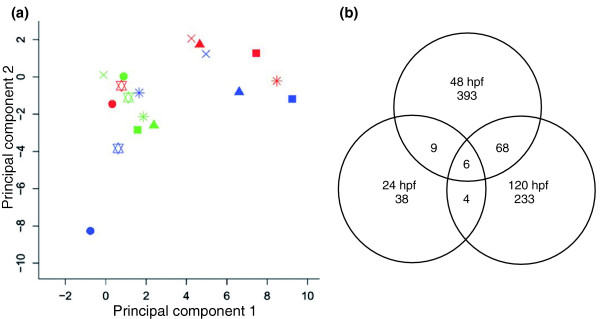
Distinct toxicogenomic expression profiles are induced by different toxicants. **(a) **Principal component analysis of the toxicogenomic profiles derived from three different embryonic stages. Embryos were exposed to vehicle controls or to one of six chemicals for the periods 4-24 hpf (green), 24-48 hpf (blue), or 96-120 hpf (red). Circles, TCDD: 150 ng/l (24 hpf), 500 ng/l (48 hpf), 500 ng/l (120 hpf). Squares, MeHg: 50 μg/l (24 hpf), 60 μg/l (48 hpf), 60 μg/l (120 hpf). Triangles, VA: 15 mg/l (24 hpf), 50 mg/l (48 hpf), 50 mg/l (120 hpf). Crosses, 4CA: 15 mg/l (24 hpf), 50 mg/l (48 hpf), 50 mg/l (120 hpf). Asterisks, Cd 500 μg/l (24 hpf), 5 mg/l (48 hpf), 5 mg/l (120 hpf). Stars, DDT: 5 mg/l (24 hpf), 15 mg/l (48 hpf), 15 mg/l (120 hpf). While the transcriptional profiles of the 4-24 hpf treatment group (green symbols) cluster closely, characteristic gene-expression profiles were induced by the 24-48 hpf (blue symbols) and the 96-120 hpf (red symbols) exposures to each of the different toxicants. **(b) **Venn diagram comparing the number of genes induced at the three stages by all six toxicants. Numbers indicate numbers of regulated or co-regulated genes at the different stages (more than 1.95-fold change and adjusted *p*_adj _< 0.025).

To assess possible stage-specific differences, we analyzed and compared the toxicogenomic response to six compounds - MeHg, Cd, 4CA, DDT, TCDD, and VA - at the three different stages. We treated several hundred embryos with each of these compounds at each of the three stages (see Materials and methods and Additional data files 1). Principal component analysis (PCA) revealed distinct toxicogenomic responses to exposure with the six toxicants in the 24-48 and 96-120 hpf treatment groups (Figure 2a). Principal components were derived by singular value decomposition (SVD). SVD is based on the decomposition of the gene-expression matrix, whose entries are the log-transformed fold changes (M values) of gene expression, into unique orthonormal superpositions of genes and treatments. Expression changes of at least twofold and a *p*_adj _< 0.025 were taken into account. The *p*_adj_-value was adjusted for multiplicity testing by controlling the false discovery rate [27].

The differences between the transcriptional profiles induced by the six toxicants were less prominent in the datasets from the 4-24 hpf treatment groups (see Figure 2a). This may be due to the fact that different toxicants caused similar gene effects at 24 hpf. For example, the expression of the gene for fast muscle troponin T (BE693169) was downregulated by Cd, MeHg, TCDD, and VA in embryos treated between 4 and 24 hpf but not at later stages (data not shown). Furthermore, many genes that are involved in organ physiology may not yet be responsive by 24 hpf, as organ development has not proceeded far enough. In agreement with this, the expression levels of only 57 genes were significantly altered by the 4-24 hpf treatment. In contrast, the expression levels of 476 and 311 genes were significantly affected by the 24-48 hpf and 96-120 hpf treatment regimens, respectively (see Figure 2b). Moreover, very few genes in the 4-24 hpf treatment set overlapped with the 24-48 and 96-120 hpf treatment groups (15 and 10 genes, respectively). The latter groups (24-48 h and 96-120 hpf) shared more gene responses (74 genes) but 393 and 233 gene responses were stage specific (see Figure 2b). The smaller number of affected genes in the 4-24 hpf regimen may also have been caused by the lower concentrations of toxicants that we had to apply to ensure sufficient survival at these younger stages. Irrespective of this, these data indicate a high stage specificity of the toxicogenomic effects in the three treatment windows.

### The toxicogenomic responses triggered by different toxicants are highly specific

**Figure 3:**
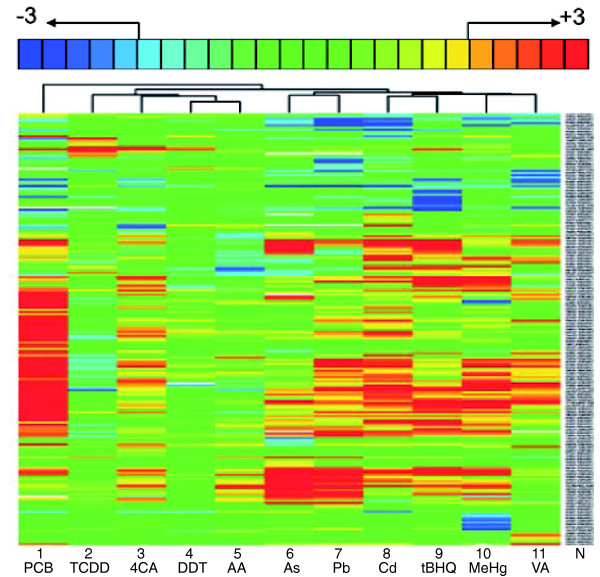
Toxicants induce highly specific toxicogenomic profiles. Hierarchical clustering of gene responses in embryos treated between 96 and 120 hpf with PCB (33 mg/l), TCDD (500 ng/l), 4CA (50 mg/l), DDT (15 mg/l), AA (71 mg/l), As (79 mg/l), Pb (2.8 mg/l), Cd (5 mg/l), tBHQ (1.7 mg/l), MeHg (60 μg/l), VA (50 mg/l). For each toxicant exposure, vehicle controls were carried out in parallel. The gene names are indicated (N) and are legible upon magnification of the PDF version of this figure. The key at the top indicates the color code for fold changes ranging from threefold upregulated (+3, red) to threefold downregulated (-3, blue). Fold changes greater than three are not indicated explicitly but are included. Only genes are listed whose mRNA levels changed by more than twofold (*p*_adj _< 0.025) in at least one of the treatments. The data represent the average over all biological and technical repeats (see Table 1).

We focused further analysis on the 96-120 hpf stage and used the full set of 11 toxicants by including treatments with AA, PCB, As, tBHQ and Pb. Replicate hybridizations with mRNA from at least three independent toxicant treatments were performed (see Table 1). Toxicant effects were clustered based on their Euclidean distance to each other and the similarity of gene responses was determined by a Pearson correlation proximity measure. The expression profiles summarize clustering results for a subset of 199 genes across all 11 toxicant responses (Figure 3). The gene-selection criteria applied take into account the extent and significance of changes in gene expression (at least twofold, *p*_adj _< 0.025) as well as differences and similarities in expression changes between toxicants (see Materials and methods). Distinct patterns of gene expression were noted for each of the 11 compounds. However, similarities in gene responses were also detected. One group of chemicals with related gene responses comprises Pb, As, Cd, tBHQ, MeHg and VA (see Figure 3, lanes 6 to 11). Another subgroup of related responses was induced by TCDD, 4CA, DDT and AA (see Figure 3, lanes 2-5), whereas the PCB triggered a more distinct expression profile (see Figure 3, lane 1).

As verification, we carried out blind tests to identify the chemicals by their induced gene-expression profile. Fourteen out of the 15 chemicals were unambiguously identified (Table 2). In the case of 4CA, close matches were scored to the 4CA, the DDT and the AA response profiles (see Table 2). Thus, we identified the correct group of chemicals (see Figure 3, lanes 2-4). Taken together, the results from these blind trials underscore the reliability of the toxicogenomic profiles and furthermore suggest that it is possible to derive signatures of toxicogenomic responses predictive for specific chemicals or chemical groups from whole animal exposure experiments.

**Table 2:** Summary of results from blind experiments

Test	4CA	Cd	DDT	MeHg	TCDD	VA	AA	As	Pb	PCB	tBHQ
Pb	6.15	6.78	8.66	6.76	10.91	7.96	7.62	6.32	**5.01**	10.66	6.81
DDT	6.74	9.86	**4.51**	8.79	7.79	8.01	5.39	10.38	8.96	10.05	9.13
4CA	6.87*	10.96	6.69*	9.47	8.82	8.87	6.99*	11.21	8.42	10.75	9.42
TCDD	8.55	12.43	6.78	11.01	**5.42**	10.37	7.61	10.76	12.61	15.63	10.62
As	9.14	9.56	10.93	9.74	12.46	9.89	10.18	**6.88**	7.51	11.32	7.51
Pb	7.25	7.95	10.05	7.38	12.06	8.62	8.65	9.58	**4.22**	9.91	7.70
PCB	9.84	9.30	13.17	10.23	15.01	10.01	11.78	12.77	10.62	**3.93**	10.40
As	9.18	9.99	9.92	10.20	11.53	10.16	9.43	**5.48**	7.84	12.40	7.67
TBHQ	8.79	7.49	11.32	9.24	11.52	10.29	10.98	8.93	9.77	11.31	**6.33**
PCB	8.37	7.74	12.06	8.88	13.65	8.54	10.63	11.79	10.22	**5.71**	9.08
AA	7.18	10.79	4.29	9.28	7.21	8.63	**3.56**	7.67	8.12	13.04	8.86
TBHQ	7.49	7.26	11.22	8.30	11.66	9.00	10.40	9.57	9.58	10.47	**5.41**
Cd	8.32	**6.67**	10.76	8.39	12.79	8.90	10.16	10.89	6.96	8.05	8.15
VA	6.43	8.25	6.47	7.57	9.04	**4.57**	6.15	9.56	8.02	10.24	8.33
AA	10.26	14.38	7.09	12.23	9.61	11.59	**6.25**	11.26	10.54	13.89	11.84

Embryos were exposed from 96 to 120 hpf with the test compounds (Test). The table gives the Euclidean distances between the expression profiles for test compounds and the 11 toxicants. Bold type indicates the correct match. An ambiguous score was obtained in the case of 4CA (*) with three closely matching expression profiles. Note that the assessment of each test compound is based on one experiment.

The induced genes fell into different gene ontology groups such as genes involved in combating oxidative stress (Table 3) and genes encoding chaperones (Table 4). Another major class of genes that was significantly regulated by a number of toxicants comprised solute carriers (Table 5). We also carried out a computational analysis of the affected genes using the GoTreeMachine algorithm to identify more complex pathways and processes (Additional data files 2-9). An inflammatory response was induced by several compounds (As, 4CA, Cd, MeHg, Pb, PCB and tBHQ), whereas inductions characteristic of an immune response were evoked by MeHg and tBHQ. The latter compound also triggered genes involved in G-protein-coupled signaling and phototransduction. Induction of genes with a function in base-excision repair was noted in the case of exposure to As and PCB, suggesting that these compounds cause DNA damage in the embryo.

**Table 3:** Oxidative stress genes and their response to toxicants

Gene name	Gene ID	AA	As	4CA	Cd	MeHg	Pb	PCB	tBHQ	VA
Peroxiredoxin 1	BI980610	3.9	13.6	4.1	3.1	7.7	9.9		7.5	
Thioredoxin	BI864190		14.2	3.5	4	4.4	8.1	2.5	6.1	
Glutathione *S*-transferase omega 1	AW019036	3	6.3	2.1		2.6	3.5		2.2	
Glutathione *S*-transferase pi	AF285098	2	4.1			3.2	5.9		2.7	
Glutathione *S*-transferase omega 2	BI979918		5.6		2.7		5.2		2.1	
Thioredoxin interacting protein	BI892352				2					2
Glutathione peroxidase	AW232474					-4.2				

Gene ID refers to the accession number in GenBank.

**Table 4:** Chaperone genes and their response to toxicants

Gene name	Gene ID	As	4CA	Cd	Pb	PCB	tBHQ	VA
Stress protein HSP70	AB062116	10.1	2.2	7	2.5	5.4	12.3	2.7
	AF210640	10.9		6.6	2.5	5	10.8	2.7
Hsp70 (2)	AF006007	7.6		6.3	2.1	4.6	10.1	2.4
Heat shock protein HSP 90-alpha	AF068773	2.6						
Heat shock cognate 70 kDa protein	BM024785	3						
DnaJ (Hsp40) homolog, family A, member 1	BI891737	3.6					3.6	
Ahsa1 protein	BM103957	2.2					2.1	

**Table 5:** Solute carrier family genes and their response to toxicants

Gene name	Gene ID	As	4CA	Cd	MeHg	Pb	PCB	VA
Solute carrier family 16 member 9 (1)	BE016639		2.5	2.9	2.4	3.6	4.1	2.3
Solute carrier family 16 member 9 (2)	BI474827		3.4	4.3	4.8	4.7	7.9	3
Solute carrier family 16 member 6	AW421040				2.9	4	4.6	2.9
Solute carrier family 2 member 5	AI477656	2.1				2.1	2.5	
Solute carrier family 6 member 8	BI980828			2		2.2	2.4	
Solute carrier family 43, member 2	BI887324						2.1	
Solute carrier family 3	BG985518						2.1	
Solute carrier family 20 (phosphate) member 1	BI890772						2.2	
Solute carrier family 6 (GABA) member 1	BF157011							-3.1
	BI563084							-2.1

### Identification of tissue-specific genes

**Figure 4:**
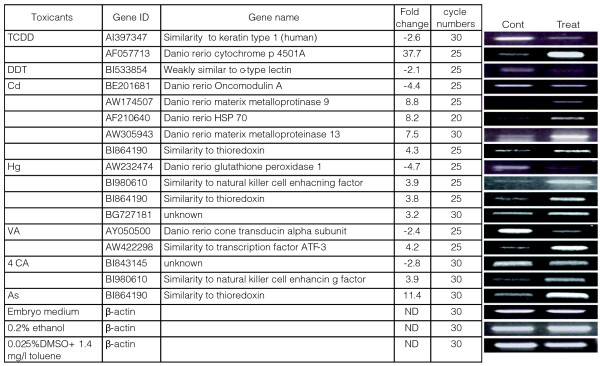
RT-PCR analysis confirms selected gene responses. Embryos were exposed to the indicated toxicants (500 ng/l TCDD; 15 mg/l DTT; 5 mg/l Cd; 60 μg/l MeHg; 50 mg/l VA; 50 mg/l 4CA; 79 mg/l As) or vehicle alone (embryo medium or 0.2% ethanol or 0.025% DMSO, 1.4 mg/l toluene) between 96 and 120 hpf. **(a) **cDNA was synthesized and subjected to PCR with primers specific for the selected genes indicated. Gene ID refers to the accession number in GenBank. The number of temperature cycles (cycle numbers) for every set of amplifications is indicated. The fold-change column summarizes the results from the microarray experiments for comparison with the RT-PCR results shown in **(b)**. See legend of Figure 1 for details of treatments and controls. β-actin mRNA was used as a toxicant-insensitive reference. ND, not determined, as the actin gene response fell into the class of nonregulated genes in the microarray results.

We next verified the observed gene responses by methods other than microarray hybridization. First, the changes in gene expression were confirmed by re-evaluating a subset of gene responses by semi-quantitative reverse transcription PCR (RT-PCR). Out of 14 gene responses analyzed, all showed the up- or downregulation expected from the array data (Figure 4). This suggests that the changes in transcript levels measured by the microarray hybridizations reflect genuine responses to the toxicants.

**Figure 5:**
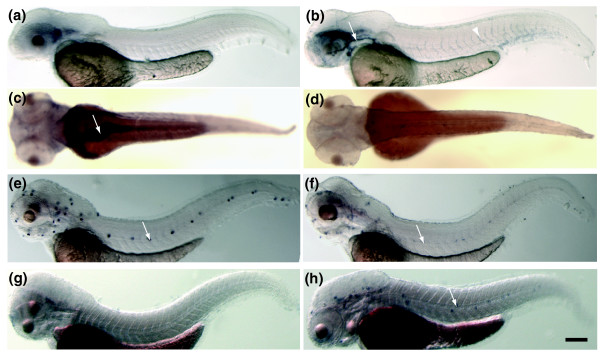
Examples of toxicant-responsive genes that are expressed in a highly tissue-restricted manner. **(a) **48 hpf vehicle 3 control. Figure 1 indicated the exposure embryo from 96 to 120 hpf and **(b) **500 ng/l TCDD-treated embryos hybridized to a cytochrome P450 1A1 antisense probe. TCDD-treated embryos showed increased levels of cytochrome P4501A1 mRNA in blood vessels Arrow, primary head sinus, arrowhead, intersegmental vessel. **(c) **72 hpf vehicle control 1 and **(d) **60 μg/l MeHg-exposed embryos hybridized to a glutathione peroxidase 1 probe. Embryos showed a reduction of mRNA levels in the gut (arrow). Embryos were treated from 4 to 72 hpf and were then fixed for *in situ *processing. **(e) **Control embryo and **(f) **500 μg/l CdCl_2_-treated embryo hybridized to oncomodulin A antisense mRNA. Oncomodulin A mRNA levels are downregulated in the hair cells of the lateral-line organ (arrow) in response to Cd exposure. **(g) **Control and **(h) **Cd-treated embryos hybridized to a thioredoxin antisense probe. Thioredoxin is upregulated in the hair cells of the neuromasts (arrow). Embryos are oriented anterior to the left and dorsal up (a,b,e-h) or with dorsal side facing (c,d). Scale bar represents 220 μm.

We used *in situ *hybridization with selected probes to toxicant-treated and control embryos to assess the tissue-specific expression patterns of the response genes and whether these are altered in response to toxicant. Cytochrome P4501A1 mRNA (AF057713) was induced by 500 ng/l TCDD in endothelial cells (15/15 embryos, Figure 5a,b). The levels of glutathione peroxidase 1 (AW232474) mRNA in stomach and gut were repressed by 60 μg/l MeHg (11/15 embryos, Figure 5c,d), in agreement with microarray and RT-PCR data (see Figure 4).

The neuromasts of the zebrafish lateral line are very sensitive to a number of compounds including CdCl_2 _[28-30]. the mRNA for oncomodulin A (also called parvalbumin3a), which is expressed in the hair cells and supporting cells of neuromasts in untreated embryos, is barely detectable in the neuromasts of embryos treated with 500 μg/l CdCl_2 _(13/15 embryos, Figure 5e,f), in concordance with the Cd-induced, 4.4-fold decrease of oncomodulin A mRNA measured by microarray hybridization (see Figure 4). In contrast, thioredoxin-like mRNA (BI864190) is upregulated in hair cells (12/13 embryos, Figure 5g,h) in response to Cd. This suggests that Cd does not cause a complete loss of hair cells, even though staining with the dye DASPEI suggests that hair cells are strongly reduced (data not shown). The thioredoxin-like mRNA is also expressed in selected areas of the brain. These regions show also increased levels of expression in response to Cd (data not shown). In summary, these *in situ *expression studies show that the microarray procedure used permits detection of organ- and cell-specific gene responses with very high sensitivity. Moreover, these results also suggest that the gene responses occur in almost all of the embryos exposed to the toxicants.

### The genome responds to very low toxicant concentrations

**Figure 6:**
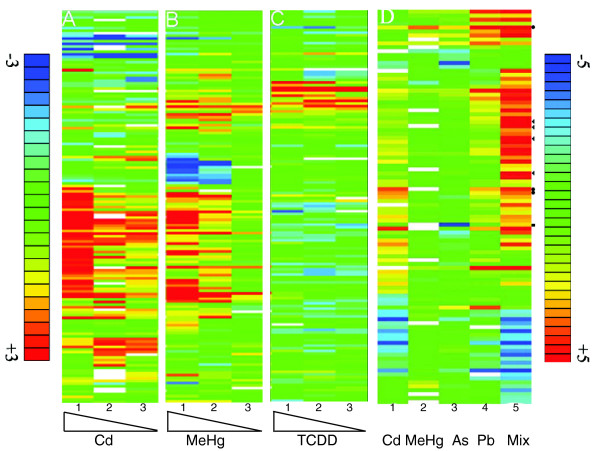
The concentration dependence of toxicogenomic responses and the synergistic effects of low doses. **(a-c) **Embryos were exposed to decreasing concentrations of Cd (a, lane 1, 5 mg/l: lane 2, 2.5 mg/l; lane 3, 0.5 mg/l), or MeHg (b, lane 1, 60 μg/l; lane 2, 30 μg/l; lane 3, 6 μg/l) or TCDD (c, lane 1, 500 ng/l; lane 2, 250 ng/l; lane 3, 50 ng/l). The low concentrations elicit significant changes in gene expression. **(d) **Embryos were exposed either to 50 μg/l Cd (lane 1) or 6 μg/l MeHg (lane 2) or 7.9 mg/l As (lane 3) or 280 μg/l Pb (lane 4) alone, or to a mixture (Mix, lane 5) of Cd (50 μg/l), Pb (280 μg/l), MeHg (6 μg/l) and As (7.9 mg/l). The mixture shows a strongly increased response with respect to the degree of changes of expression of individual genes (dark red and dark blue bars). Arrowheads point to examples of synergistic responses whereas the dots highlight genes whose response seems to be additive. The square indicates a gene that was downregulated by As and slightly upregulated by the mixture. All exposures were performed between 96 and 120 hpf. The color key for fold changes in gene expression in (a-c) is indicated on the left and ranges from threefold upregulated (red) to threefold downregulated (blue). The color key for (d) is on the right and ranges from fivefold upregulated (red) to fivefold downregulated (blue). White bars indicate missing data. Only genes were listed whose mRNA levels changed by at least twofold (*p*_adj _< 0.025) in at least one of the treatments. The data represent the average over all biological and technical repeats (see Table 1).

The concentrations of the toxicants were adjusted in the initial experiments so that they caused morphologically visible defects in exposed animals. We asked next whether one could measure changes in the expression profiles at lower concentrations that do not have apparent morphological effects. TCDD, DDT, Cd, 4CA, MeHg, and VA were used as a set of test compounds. We could detect significant changes in gene expression (at least twofold and *p*_adj _< 0.025) in response to 0.5 mg/l Cd, 6 μg/l MeHg, 5 mg/l VA, 25 mg/l 4CA, 15 mg/l DDT, and 50 ng/l TCDD (Figure 6a-c, Table 6, and data not shown). With the exception of 6 μg/l MeHg and 25 mg/l 4CA, these low concentrations did not cause obvious morphological or behavioral defects (data not shown), suggesting that this assay can detect responses to toxicant concentrations that do not cause acute morphological effects. It is clear, however, that the number of genes with a significant response to the toxicants decreases (see Table 6). Cytochrome P4501a1 was fivefold upregulated by 50 ng/l TCDD, oncomodulin A was reduced 4.5-fold by 0.5 mg/l Cd and peroxiredoxin was still 3.5-fold induced by 6 μg/l MeHg. Thus, even though fewer genes respond to these lower concentrations, the measured changes in transcript levels are robust.

**Table 6:** The number of regulated genes in response to different concentrations of toxicants

Toxicants	Stage	Concentration	Number of regulated genes
4CA	120 hpf	50 mg/l	201
		25 mg/l	2
		5 mg/l	0
Cd	120 hpf	5 mg/l	475
		2.5 mg/l	102
		0.5 mg/l	57
DDT	120 hpf	15 mg/l	25
		1.5 mg/l	0
		0.15 mg/l	0
TCDD	120 hpf	500 ng/l	34
		250 ng/l	34
		50 ng/l	4
VA	120 hpf	50 mg/l	335
		25 mg/l	1
		5 mg/l	4
MeHg	120 hpf	60 μg/l	417
		30 μg/l	20
		6 μg/l	9

The fold change was equal to or greater than 1.5 and *p*_adj _< 0.025. Note that for reasons of increased sensitivity we used a lower fold-change cutoff in this experiment compared with that in Figure 2b, which explains the higher number of regulated genes in this set of experiments.

### Complex synergistic effects are evident in toxicogenomic responses to compound mixtures

In the environment we are normally confronted with compound mixtures rather than pure substances. The components of these mixtures could act synergistically, thereby potentiating the toxic effect [31]. We therefore investigated whether synergistic effects of compound mixtures can be observed in toxicogenomic profiles. To this end, 96-hpf embryos were exposed to a mixture of low concentrations of Cd (50 μg/l), Pb (280 μg/l), MeHg (6 μg/l) and As (7.9 mg/l). About twice as many genes (158 genes) were significantly up- or downregulated (absolute change at least twofold, *p*_adj _< 0.025) than the sum of the genes regulated by exposure to the individual toxicants (81 genes: Cd 48 genes; As 12 genes; MeHg 5 genes; Pb 16 genes). Complex expression profiles composed of both additive and synergistic as well as novel patterns of gene responses (at least twofold change, *p*_adj _< 0.025; Figure 6d) were scored for the mixture. In the case of the genes with similarity to peroxiredoxin (BI980610) or the solute carrier family members 6 and 9 (BE016639, AW421040), the response to the mixture appears to be purely additive (see Figure 6d, dots; Table 6, and Additional data file 13). In other instances, for example the Hsp70-related genes (AB062116, AF210640, AF006007) or the sequestosome1 gene (AW343560), the mixture induced a strong increase in transcript levels, whereas significant gene responses (more than twofold, *p*_adj _< 0.025) were not induced by administration of the individual compounds (see Figure 6d, arrowheads, Table 6, Additional data file 13). These genes can, however, be induced by higher concentrations of the individual compounds (see Figures 3, 6a,b and Additional data files 10, 11), suggesting that the observed synergy is the result of a lowered response threshold. Curiously, we also noted loss of gene responses on exposure to the compound mixture (see Figure 6d), suggesting suppressive effects of the combination. For example, the transcript levels of glutathione-*S*-transferase omega 1 (AW019036) are significantly altered by exposure to PbCl_2_, but not by the mixture (see Table 3 and Additional data file 13). In a few instances we observed opposing effects, such as in the case of suppressor of cytokine signalling 3 (BI878700), which was 4.9-fold downregulated by As and 2.6-fold upregulated by the mixture (see Additional data file 13). Taken together, these results show potentiated, additive, and nonadditive effects of the mixture in comparison to the individual compounds.

## Discussion

We have shown that a diverse set of 11 chemicals induces highly specific gene responses in the zebrafish embryo. Moreover, synergy effects and responses to low-dose exposure were detectable in the genome-wide transcriptional response. Our work provides proof of principle that the zebrafish embryo can serve as a specific and highly sensitive whole-animal model to monitor the toxicogenomic impact of chemicals.

Although vertebrate cell lines and other *in vitro *test methods have great merits in assessing toxicological effects of drugs and pollutants, they cannot replace whole animal test systems entirely. The classical animal models such as mice, rats and rabbits are expensive and attract concerns from animal-rights groups. Zebrafish embryos before the feeding stage offer a cheap and ethically acceptable vertebrate model that will not only be useful in the toxicological assessment of the tens of thousands of compounds to be tested under the REACH program but can also help to evaluate the developmental toxicity of novel compounds at an early stage of drug development.

The requirement for adequate animal models for assessing developmental toxicology is further underscored by the remarkable stage dependence of the observed toxicogenomic profiles. These differences in gene responses are likely to be a reflection of the dynamics of cell differentiation and morphogenesis, which will be impossible to model in all their aspects in cell culture and other *in vitro *systems. The differences in gene responses were particularly striking at early stages, presumably reflecting the fact that many organs exist only as rudiments at these times and have not fully acquired their physiological function. It is also possible that the inter-embryonic variability of the gene responses is higher at this stage, blurring the gene-expression changes in the pooled cDNA.

Previous work showed that the sensitivity of the zebrafish embryo to toxicants equals that of the commonly used tests on adult freshwater fish, allowing a reliable prediction of the toxic potential of chemicals [10,11]. The embryonic DarT assay [10,11] uses an exposure paradigm from cleavage stages to 48 hpf and relies on a set of morphological endpoints and lethality. Morphological readouts provide little discrimination between the effects of different compounds, especially in the case of environmental toxicants with a broad spectrum of toxic effects on the embryo. In marked contrast to the morphological endpoints, we found highly specific patterns of transcriptional changes, resulting in barcode-like patterns of gene responses. With one exception, we were able to predict the chemical unequivocally by its pattern of induced gene-expression changes. In most cases, these patterns are related, forming distinct subgroups of profiles, but are still sufficiently different from one another to discriminate the individual compounds.

Strikingly, a general response to oxidative stress or protein damage does not seem to exist in the zebrafish embryo. A number of the chemicals (see Table 3) induced genes involved in the cellular systems that combat the effects of oxidative stress [32]. However, the induced oxidative-stress genes differed between chemicals, suggesting toxicant-specific effects (see Table 3). A similar observation was made with respect to chaperones (see Table 4). The tissue-specific expression of these genes as well as restricted tissue effects of the toxicant may be important in this context. For example, the expression of the thioredoxin-like gene is restricted to a small number of neurons in the brain. In *in situ *hybridization experiments, strong elevation of thioredoxin-like mRNA levels in response to Cd and MeHg was also noticed in the hair cells of the lateral line as well as in the brain. The differences in the type of induced defense genes and their tissue-restricted expression suggest tissue-specific effects of the different toxicants.

Another Gene Ontology (GO) group that is differentially regulated by exposure to a number of toxicants is represented by members of the solute carrier (SLC) family (Table 5). These transmembrane proteins have key roles in the transport of small molecules including neurotransmitters across vesicular and plasma membranes [33]. It is tempting to speculate that the specific downregulation of the GABA transporter SCL6 member 1 by VA (see Table 5) may be related to the therapeutic effect of VA as a suppressor of epileptic seizures.

The concentrations that elicited toxicogenomic responses are in the range of pollutant levels prevailing in the environment. We did not, however, exclude the possibility that compounds accumulate in the embryo, resulting in higher intra-embryonic concentrations than in the environment. Toxicogenomic responses were triggered by TCDD, Cd, DDT, and VA at concentrations that did not cause changes in morphology. Thus the genomic response appears to be more sensitive to toxic insult than is morphogenesis. A crucial question is whether the gene responses that are not obviously correlated with pathological alterations are indeed deleterious to the animal. For example, TCDD was shown to induce a battery of genes in the mouse paw (including homologs of genes we scored in our study) without obvious teratological consequences to paw development [34]. Future work will need to address whether the low-level effects on gene expression could be correlated with, and hence used to predict, chronic effects of long-term exposure.

The lowest concentration of MeHg (6 μg/l) triggered significant changes in gene expression. In addition, we also noted teratological effects on movement and tail development at these concentrations (L.Y. and J.R.K., unpublished work), indicating that low concentrations of MeHg are acutely toxic in the zebrafish embryo. Disturbingly, blood serum levels of MeHg in humans can be in the same concentration range [35]. The zebrafish embryo may be much more susceptible to MeHg, but defining blood serum levels that are regarded as safe in humans is an active area of research.

Application of a mixture of MeHg, Cd, As, and Pb at low concentrations resulted in synergistic effects with more than additive numbers of genes affected and also novel patterns of gene-expression changes. Clearly, some of the genes affected by exposure to the mixture would be induced or repressed by higher concentrations of the individual chemicals. Examples are the thioredoxin and Hsp70 genes. Thus, it appears that the threshold at which induction occurs is lowered. This agrees with previous studies of mixture effects that support the notion of 'concentration addition', in which a component of the mixture can be replaced by an equipotent concentration of another compound [31]. The patterns of gene-expression changes induced individually by the four chemicals differed, however, suggesting that other effects have to be taken into account that cannot be explained by an additive mechanism of action.

Expression levels of genes, and presumably also responses to environmental toxicants, can vary dramatically between individuals. In a systematic study of variation in gene expression in natural populations of fish of the genus *Fundulus*, significant differences in gene expression were noted in 18% of the 907 genes analyzed [36]. In this respect, zebrafish embryos have a big advantage over mammalian systems as one can easily obtain large numbers of embryos and can thus average the individual gene responses by using pooled cDNA prepared from many embryos. In the cases where we confirmed the gene responses by *in situ *hybridization, we found that most individuals showed the expected upregulation, suggesting that many of the observed responses have a high penetrance.

While the complete development outside of the mother and the transparency of the zebrafish embryos are certainly important advantages for observation, the small size of the embryos limits the possibility of dissecting particular organs for toxicogenomic analysis. To overcome these limitations, one can use transgenic animals expressing green fluorescent protein and fluorescence-activated cell sorting to enrich for particular cell types [37]. Moreover, even whole-embryo exposure protocols as we used here permit detection of highly tissue-restricted gene responses such as those seen, for example, in the lateral line, which comprises only a very small fraction of the whole embryo.

## Conclusion

The induction of the Hsp70 gene was previously shown to be a sensitive biomarker in zebrafish for exposure to Cd and other heavy metals [38]. The work presented here adds a long list of other highly sensitive biomarkers to be developed as transgenic biosensors. We believe that the zebrafish could become a key model for molecular developmental toxicology. Functional studies of TCDD toxicity in the zebrafish embryo well illustrate this (reviewed in [39]). Forward genetics [40-43], targeting-induced local lesions in genomes (TILLING) [40], morpholino knockdown [44], transgenesis [38,45] and *in situ *expression studies [46,47] at cellular resolution represent a powerful technical repertoire for dissecting toxicological pathways. Moreover, a large number of developmental mutants have been isolated, some of which may serve as direct targets for drugs and toxicants [48,49]. We believe that the work on the toxicogenomics of zebrafish embryos reported here is a fundamental contribution to the use of the zebrafish embryo as a model system for molecular developmental toxicology.

## Materials and methods

### Chemicals and embryo treatment

AA (acrylamide; CH_2 _= CHCONH_2_), PCB (Aroclor 1254), As (arsenic (III) oxide; As_2_O_3_), tBHQ (tert-butylhydroquinone; (CH_3_)_3_CC_6_H_3_-1,4-(OH)_2_), Cd (cadmium chloride; CdCl_2_2H_2_O), 4CA (4-chloroaniline; ClC_6_H_4_NH_2_), DDT (1,1-bis-(4-chlorphenyl)-2,2,2-trichlorethane; ClC_6_H_4_)_2_CHCCl_3_), Pb (lead (II) chloride; PbCl_2_), MeHg (methylmercury chloride; CH_3_ClHg), TCDD (2,3,7,8-tetrachlorodibenzo-p-dioxin) and VA (valproic acid; CH_3_(CH_2_)_4_CO_2_H) were purchased from Sigma-Aldrich (St Louis, MO).

Wild-type zebrafish strains AB, ABO and Tübingen were kept and bred as described [50]. Embryos were grown in embryo medium (60 μg/ml Instant Ocean, Red Sea, Houston, TX). Different numbers of embryos were exposed to the chemicals: 4-24 hpf (600 embryos), 24-48 hpf (400 embryos) and 96-120 hpf (200 embryos). Vehicle controls used were embryo medium alone (for Cd, Hg, Pb, As, VA, AA treatments) or 0.2% ethanol in embryo medium (for tBHQ, 4CA, PCB, DDT treatments) or 0.025% DMSO, 1.4 mg/l toluene in embryo medium (500 ng/l TCDD) or 0.0075% DMSO, 420 μg/l toluene (150 ng/l TCDD) or 0.0025% DMSO, 140 μg/l toluene (50 ng/l TCDD). Toxicant concentrations were adjusted in such a way that embryo death was minimal. The few dead embryos were discarded before preparation of RNA. None of the vehicle controls had an apparent toxic effect on the embryos by itself. As the toluene-related chemical benzene can synergize with TCDD [51] we cannot completely exclude a synergistic effect between TCDD and toluene at the 1.4 mg/l toluene concentration.

### Microarray analysis

A total of 16,399 gene-specific 65mers designed by Compugen (Jamesburg, NY) and produced by Sigma-Genosys (The Woodlands. TX) were purchased and the probes (40 mM) were spotted in duplicate in two separate subarrays using a Gene Machines Omnigrid 100 (San Carlos, CA) and TeleChem SMP3 pins (Sunnyvale, CA) on CodeLink activated slides (GE Healthcare, Chalfont St Giles, UK). Upon evaluation it turned out, however, that plates 29 to 43 had faulty amine linkers, impairing the retention of the oligonucleotides on the coat-link slides. As the companies were unable to replace the defective oligonucleotides, we used the reduced set of intact oligonucleotides (384-well plates 1 to 28).

Total RNA was isolated from toxicant- and vehicle-treated embryos in every experiment in parallel using the Nucleospin RNA L Kit (Macherey-Nagel, Düren, Germany) and mRNA was extracted with the Ambion Purist Kit (Austin, TX). Labelled cDNA was synthesized from 1-2 μg mRNA using the Amersham direct cDNA labeling kit (Amersham Europe, Freiburg, Germany). Upon removal of unincorporated nucleotides over Microcon 30 spin columns (Millipore, Bedford, MA), the concentrated probes were hybridized to the microarray in 1× DIG Easy-Hyb buffer (Hoffmann-La Roche, Basel, Switzerland) overnight at 42°C. Coverslips were removed from the slides by flushing with 4× SSC and slides were washed in prewarmed wash buffer 1 (2× SSC, 0.1% SDS) for 5 min at 42°C, then in buffer 2 (0.1× SSC, 0.1% SDS) for 10 min at room temperature, and finally in 0.1× SSC four times for 1 min at room temperature. The slides were briefly dipped into 0.01× SSC at room temperature before centrifugation for 7 min at 800 rpm in an Eppendorf 5810R centrifuge.

Arrays were scanned using the Axon model 4000B dual-laser scanner and the corresponding GenePix 6 software (Molecular Devices, Union City, CA). Both channels (532 nm for Cy3 and 635 nm for Cy5) were scanned in parallel and stored as 16-bit TIFF files. Each array was scanned three times (low, medium, and high scan) with different signal-amplification factors (voltage settings of the photomultiplier tubes), but with the same laser power. The channels for Cy3 and Cy5 were balanced in each scan for approximately the same intensity range. For the low scan no spot was saturated; in the high scan the signal amplification for Cy5 was set to approximately 80% of maximum and Cy3 amplification was adjusted to this. The settings used in the medium scan lie between the low and the high scan. The absolute intensity values span the range from 0 to 65536. The scans were performed with a resolution of 10 μm. From each spot with a mean diameter of 100 μm, 70-80 pixels were recorded. Individual local background areas around the spots were defined, which comprised approximately 400 pixels. For each channel, the spot signals were calculated as the median intensity of all foreground pixels minus the median intensity of all background pixels.

All microarray data from this study have been deposited in NCBI's Gene Expression Omnibus under the accession number GPL4603.

### Data preprocessing, quality control, transformation, and normalization

Raw data was derived from the result files generated by the GenePix 6 suite and analyzed with the R software [52]. Preprocessing of data comprises mapping of scans, quality control, transformation, and normalization steps. Signal intensities from low, medium, and high scans are mapped onto the same scale by an affine transformation. Transformation parameters are estimated based on a least-squares optimization. Averaging the transformed intensities gives the consensus signals, which are independent of the voltage settings of the photomultiplier tube.

Quality control was performed on a spot and array level. Spots ideally have a diameter of 100 μm. Diameters less than 70 μm and greater than 140 μm are indicative of scratches and printing problems and the corresponding data was discarded. In addition, inconsistent spots with a coefficient of variance of pixels bigger than 0.7, and weak spots with a foreground signal less than 175% of the background signal were removed from further analyses. Strong but unreliable signals with at least 20% of pixels in saturation were discarded. Quality control on array level determined the overall quality of each single chip. Therefore, results from different arrays were compared with each other on the basis of correlation parameters, scatterplots and chi-plots for all combinations of arrays for a particular treatment [53,54]. Raw intensities were transformed with the natural logarithm. A locally weighted regression smoother (LOESS) was applied to correct intensity-dependent signal patterns [55]. The regression is a first-order polynomial that takes into account the subset of 25% of spots that yield a signal with similar intensities. Variance stabilization for weakly expressing genes was not performed as such effects were not apparent. All chips hybridized for a particular treatment were scaled to a common median absolute deviation from median (MAD) of the logarithmic fold change (M value) [56]. Statistical analysis was based on the assumption that the majority of genes are not changed in their expression and that the overall up- and downregulations compensate each other in sum.

Each individual gene was tested for difference in expression under toxic conditions with a *t*-test where an adjusted *p *value (*p*_adj_) of less than 0.025 indicated significant differential expression. Statistical requirements of normal distribution and homoscedasticity are tenable. A robust variance estimation was derived by balancing gene-specific and pooled variance [57]. The number of false positives due to multiple testing was reduced by adjusting the resulting *p *values by controlling the Benjamini-Hochberg false discovery rate [27].

Multivariate analysis was based on a subset of genes of interest. Genes that remain unchanged under all conditions were ignored. Marker genes that are significantly changed by exposure to a particular toxicant were taken into account. In addition, the selected subset included genes that showed a global response across many chemicals. The selected subset included: the top 20 up- or downregulated genes based on fold change (minimum fold change > 2); the top seven genes with the highest correlation among at least two toxicants (minimum correlation > 0.7); the top 100 genes with the highest MAD across all treatments; and the marker genes that are regulated at least threefold for just one treatment.

Most multivariate approaches require a complete dataset without missing values. Under the condition that more than 80% of the data for a particular gene is available, missing data for gene *g *are imputed by a k-nearest-neighbor algorithm [58]. Missing values are estimated as weighted average of the values for the *k *genes with the closest Euclidean distance to gene *g*.

The logarithmic fold changes (M values) of genes under toxic conditions are subjected to PCA and hierarchical clustering. The principal components of experimental data across all experiments were derived by SVD [59]. Gene-expression profiles summarize clustering information for toxicants and genes. Dissimilarity between toxicants is determined as Euclidean distance of their M values. In contrast, proximity between two genes is derived as the arc cosine transformed Pearson correlation coefficient [60].

GO analysis of toxicant-affected genes was carried out by extracting the human homologs from the Zebrafish Chip Annotation Database [61]. The GO trees and categories were established with the web-based GoTreeMachine [62]. The number of genes with significant alterations in expression levels in response to TCDD, DDT, and AA were too few to be analyzed by GoTreeMachine.

### Expression analysis

*In situ *hybridization and RT-PCR were carried out using standard procedures [46,63]. The sequences of the primers used in RT-PCR are listed in Additional data file 14. Embryos and RNA samples were derived from independent toxicant exposures. Cell death was monitored by acridine orange staining and examination by fluorescence microscopy [64].

## Additional data files

Additional data is available online with this paper. Additional data file 1 summarizes the preliminary data evaluating the effectiveness of toxin treatment. Additional data file 2 contains a GO tree for arsenic oxide. Additional data file 3 contains a GO tree for 4CA. Additional data file 4 contains a GO tree for cadmium chloride. Additional data file 5 contains a GO tree for methylmercury. Additional data file 6 contains a GO tree for lead chloride. Additional data file 7 contains a GO tree for PCB. Additional data file 8 contains a GO tree for tBHQ. Additional data file 9 contains a GO tree for VA. Additional data file 10 summarizes gene responses of embryos exposed to different concentrations of Cd. Additional data file 11 summarizes gene responses of embryos exposed to different concentrations of MeHg. Additional data file 12 summarizes gene responses of embryos exposed to different concentrations of TCDD. Additional data file 13 summarizes gene responses of embryos exposed to 50 μg/l CdCl_2 _(Cd) or 6 μg/l MeHg (MeHg) or 7.9 mg/l As_2_O_3 _(As) or 280 μg/l PbCl_2 _(Pb) alone or to a mixture (Mix). Additional data file 14 is a list of primers used in the RT-PCR experiments shown in Figure 4.

## Authors' contributions

L.Y. and U.S. conceived the work and designed the experiments. U.S. supervised the project and wrote the manuscript. L.Y. performed all of the experimental work. J.R.K. optimized some of the concentrations of the toxicants. C.Z. and J.J performed statistical analysis of the microarray data. M.B. and M.P. provided technical assistance in microarray printing. F.M. gave technical advice at an early stage of the project. J.L. performed the gene ontological analysis.

## Supplementary Material

Additional data file 1 Embryos were exposed between 24 and 48 hpf and the morphological defects were scored at 72 hpf. Click here for file

Additional data file 2 Significant gene ontology groups affected by the toxicant are indicated in red. Click here for file

Additional data file 3 Significant gene ontology groups affected by the toxicant are indicated in red. Click here for file

Additional data file 4 Significant gene ontology groups affected by the toxicant are indicated in red. Click here for file

Additional data file 5 Significant gene ontology groups affected by the toxicant are indicated in red. Click here for file

Additional data file 6 Significant gene ontology groups affected by the toxicant are indicated in red. Click here for file

Additional data file 7 Significant gene ontology groups affected by the toxicant are indicated in red. Click here for file

Additional data file 8 Significant gene ontology groups affected by the toxicant are indicated in red. Click here for file

Additional data file 9 Significant gene ontology groups affected by the toxicant are indicated in red. Click here for file

Additional data file 10 Summary of gene responses of embryos exposed to different concentrations of Cd. Click here for file

Additional data file 11 Summary of gene responses of embryos exposed to different concentrations of MeHg. Click here for file

Additional data file 12 Summary of gene responses of embryos exposed to different concentrations of TCDD. Click here for file

Additional data file 13 ID, GeneBank accession number. Click here for file

Additional data file 14 List of primers used in the RT-PCR experiments shown in Figure 4. Click here for file
