# Supplementary material for: Transcriptional profiling reveals barcode-like toxicogenomic responses in the zebrafish embryo
Source: Genome Biol. 2007 Oct 25;8(10):R227. doi: 10.1186/gb-2007-8-10-r227 (PMC2246301; doi:10.1186/gb-2007-8-10-r227)
Supplement: Additional data file 9 — Significant gene ontology groups affected by the toxicant are indicated in red. [file gb-2007-8-10-r227-S9.doc]

|  | Head % | Trunk % | Yolk sac % | Edema % | Pigmentation % |
| --- | --- | --- | --- | --- | --- |
| 4CA (50 mg/l) | - | 68 | 69.2 | 55.6 | - |
| DDT (15 mg/l) | 30.2 | 65.1 | 57.3 | 28.4 | - |
| TCDD (500 ng/l) | 73.1 | 91.9 | 78.8 | 80.5 | - |
| VA (50 mg/l) | - | 65.3 | 43.1 | 55.8 | - |
| MeHg (60 µg/l) | 75.1 | 68.3 | 46.8 | 51.3 | - |
| Cd (5 mg/l) | 2.3 | 62.2 | 11.1 | 9.4 | - |
| PCB (33 mg/l) | 1 | 41.3 | - | 10 | - |
| Pb (2.8 mg/l) | 1 | 72 | 1.2 | - | - |
| AA (71 mg/l) | - | - | 10 | 10 | - |
| As (79 mg/l) | - | 1 | 4 | 6 | - |
| tBHQ (1.7 mg/l) | - | - | - | - | 100 |
| Control (embryo water) | - | 1.3 | - | 1 | - |
| VC1(0.2% ethanol) | - | 1.8 | 1 | 1 | - |
| VC2 (0.025% DMSO, 1.4 mg/l toluene) | - | 1.5 | 2 | 1.2 | - |

Yang et al, Additional data file 9
