# Supplementary material for: Transcriptional profiling reveals barcode-like toxicogenomic responses in the zebrafish embryo
Source: Genome Biol. 2007 Oct 25;8(10):R227. doi: 10.1186/gb-2007-8-10-r227 (PMC2246301; doi:10.1186/gb-2007-8-10-r227)
Supplement: Additional data file 14 — List of primers used in the RT-PCR experiments shown in Figure 4. [file gb-2007-8-10-r227-S14.doc]

Yang et al. Additional data file 14

| Gene ID | Primers |
| --- | --- |
| AI397347 | Forward: GGCAGGTCGATCTGAATCAT  Reverse: TTGACACCACTTGTCCATCG |
| AF057713 | Forward: TGGATGAAAAGATCGGGAAG  Reverse: GCCAGTTTTCCTGGATTTCA |
| BI533854 | Forward: GCAGGAATTGGAACATGTCA  Reverse: TTGCTTTGCTTTTATTGATTTGA |
| BE201681 | Forward: CGGACACCACTCAGCCTTAT  Reverse: TGTGCGCTCTCTACATGTCC |
| AW174507 | Forward: GCCCTGATCGTGGATACAGT  Reverse: TTGCCTTTTCCTCTCTGCAT |
| AF210640 | Forward: GGCACCACCTACTCCTGTGT  Reverse: CACAAAGTGGTTCACCATGC |
| AW305943 | Forward: ACCAGGAGACACTGGAGGTG  Reverse: AGGATAACTGCGCCAGAAGA |
| BI864190 | Forward: GCTGACACAGCCTGTCAATAA  Reverse: GATCGACACAAACATCCGACT |
| AW232474 | Forward: ATTTTTCGCGCCTCCTATTT  Reverse: CCCGATGAGGAACTTTTCAA |
| BI980610 | Forward: CGCGAGTTCACTTTCATTCA  Reverse: GTGTCTTTTCCAGGCTTCCA |
| BG727181 | Forward: CGATTCCTCCTCCTTTTTCC  Reverse: CTCTTGCTCCATCCTTCCAG |
| AY050500 | Forward: ACGGTCAAACTTCTGCTGCT  Reverse: AAGCGCTAAGAGCTCCACAG |
| AW422298 | Forward: GGACGTGGAGATGACAAGGT  Reverse: TCGGATGTCTTGTGGTGTGT |
| BI843145 | Forward: TTCTGCATTTCTTCCGGGTA  Reverse: ATGTTGCACTTTTGCTGACG |
| NM_131031  Β-actin 1 | Forward: CCCAGACATCAGGGAGTGAT  Reverse: AGGAAGGAAGGCTGGAAGAG |
